# Association of arsenobetaine with beta-cell function assessed by homeostasis model assessment (HOMA) in nondiabetic Koreans: data from the fourth Korea National Health and Nutrition Examination Survey (KNHANES) 2008-2009

**DOI:** 10.1186/s40557-017-0181-0

**Published:** 2017-07-10

**Authors:** Kiook Baek, Namhoon Lee, Insung Chung

**Affiliations:** 1 Division of Occupational and Environmental Medicine, Keimyung University Dongsan Medical Center, Daegu, South Korea; 2 Department of Preventive Medicine, Keimyung University School of Medicine, Daegu, South Korea

**Keywords:** Arsenic, Arsenobetaine, Pancreatic β-cell, Diabetes, Organic arsenic

## Abstract

**Background:**

Arsenic is known as an endocrine disruptor that people are exposed to through various sources such as drinking water and indigestion of marine products. Although some epidemiological and animal studies have reported a correlation between arsenic exposure and diabetes development, there are limited studies regarding the toxic effects of organic arsenic including arsenobetaine on the human body. Here, we analyzed the association between urine arsenobetaine and the homeostasis model assessment of β-cell function (HOMA-β), which is an index for predicting diabetes development and reflecting the function of pancreatic β-cells.

**Methods:**

In the fourth Korea National Health and Nutrition Examination Survey (KNHANES), health and nutrition surveys and screening tests were performed. Of the total survey population, people with confirmed values for urine total arsenic and arsenobetaine were included, and known diabetic patients were excluded. A total 369 participants were finally included in the study. We collected surveys on health, height, body weight, body mass index, blood mercury level, fasting glucose level, and serum insulin level and calculated HOMA index. Owing to sexual discrepancy, we performed sexually stratified analysis.

**Results:**

Urine total arsenic and total arsenic minus arsenobetaine was not associated with HOMA-IR and HOMA-β in univariate analysis or in sexually stratified analysis. However, urine arsenobetaine showed a statistically significant relationship with HOMA-β in univariate analysis, and only male participants showed a significant correlation in sexually stratified analysis. In the analysis adjusted for age, BMI, smoking, alcohol drinking, physical activity and blood mercury, the HOMA-β value in the group below the 25th percentile of arsenobetaine was significantly higher than the group between 50 and 75th percentile, while no difference was shown for HOMA-IR. In sexually stratified analysis, The value of HOMA-β was significantly higher in male participants with below the 25th percentile urine arsenobetaine than the group between 25 and 50th and between 50 and 75th, while no difference was shown for HOMA-IR. However, female participants did not demonstrate a relationship between HOMA–IR, HOMA-β and urine arsenobetaine.

**Conclusion:**

This study revealed the association between urine arsenobetaine and pancreatic β-cell function assessed by HOMA-β in the normal population (without diabetes), especially in males, despite adjusting for factors affecting pancreatic β-cell function and diabetes.

**Electronic supplementary material:**

The online version of this article (doi:10.1186/s40557-017-0181-0) contains supplementary material, which is available to authorized users.

## Background

Arsenic is a well-known toxic material that people can be exposed to through various sources. Exposure to inorganic arsenic is mainly through drinking water [1] and to organic arsenic is through marine products and seaweeds [2]. Arsenic is known as an endocrine-disrupting chemical causing various endocrine disorders [3].

Diabetes is reported to develop through two mechanisms: dysfunction of pancreatic β-cells and insulin resistance. The homeostasis model assessment (HOMA) model is widely accepted in epidemiological studies of pancreatic β-cell function and insulin resistance, which includes HOMA-IR for insulin resistance and HOMA-β for pancreatic β-cell function [4].

Many epidemiological and experimental studies have demonstrated the association between arsenic exposure and diabetes [5, 6]. Although there have been some speculative mechanisms of developing insulin resistance and β-cell dysfunction from arsenic exposure, the cellular and molecular mechanisms involved remain unclear [7].

Previous studies have used urine arsenic or urine inorganic arsenic as a marker of biological exposure for evaluating the correlation between arsenic, diabetes, and insulin resistance; however, organic arsenic, such as arsenobetaine, has been observed as a confounding factor [8, 9]. Although previous studies have reported arsenobetaine as the least toxic agent that is almost not absorbed and not metabolized in the body and excreted [10], recent studies have revealed that arsenobetaine could accumulate in human body or transform into toxic inorganic arsenic in the gastrointestinal tract by microorganisms [11, 12]. There have been no studies on the relationships among arsnenobetaine and pancreatic β-cell function.

This study aimed to reveal the effect of arsenobetaine on pancreaic β-cell function by analyzing the association among HOMA index and urine arsenobetaine of non-diabetes population using original data from the Korea National Health and Nutrition Examination Survey (KNHANES) performed in 2008 − 2009.

## Methods

### Population

This cross-sectional study used original data from the fourth KNHANES performed in 2008 − 2009. The survey was conducted by the Korean Ministry of Health and Welfare to investigate the health and nutrition status of Koreans, which included health and diet surveys as well as laboratory tests. In the fourth KNHANES, a total of 24,871 participants were enrolled. Of these participants, measurement of urine arsenic and speciation analysis of arsenic species of 202 and 200 participants were performed in 2008 and 2009, respectively. Known diabetic patients (*n* = 23) and participants with a fasting glucose level of over 126 mg/dL (classified as diabetic patients, *n* = 8) were excluded. Participants with missing data or who answered ‘Don’t know’ in the survey for smoking, drinking, and exercise were also excluded (*n* = 2). Finally, 369 participants were included in the study.

### Blood sample

All blood samples were analyzed within 24 h after sampling. Plasma glucose was measured by Hitachi Automatic Analyser 7600 (Hitachi, Tokyo, Japan). Serum insulin was analyzed by immunoradiometric assay with 1470 WIZARD Gamma Counter (PerkinElmer, Turku, Finland). Blood mercury concentrations were measured by gold amalgam method (DMA-80, Milestone, Italy). The minimal detection limit of blood mercury was 0.05 μg/L.

### HOMA-IR and HOMA-β percentage

HOMA-IR and HOMA-β percentage was calculated using the formula: HOMA-β (%) = [360 × fasting insulin (mIU/L)] / [ fasting glucose (mg/dL) – 63 ], HOMA-IR=[fasting glucose (mg/dL) × fasting insulin (mIU/L)]/405 [4].

### Speciation analysis of urine arsenic

Speciation analysis of urine arsenic was performed by inductively coupled plasma-mass spectrometry (ICP-MS) with ELAN DRC-e (PerkinElmer, Turku, Finland), adjusted for creatinine. The reagents used were Arsenic Standard solution (Sigma-Aldrich, MO, USA), Triton X-100 (Sigma-Aldrich, MO, USA), and concentrated nitric acid (Dongwoo Fine-Chem, Iksan, Korea). The minimum detection limit of arsenobetaine was 0.11 μg/L. Values below the limit of detection was estimated as 1/2 limit of detection [13, 14].

### Statistical analysis

Categorical data are expressed as numbers of cases and percentages, and continuous data are expressed as means ± standard deviation. Urine arsenic and arsenobetaine did not follow a normal distribution, so the values are expressed as the median, 25th percentile, and 75th percentile. The index for smoking, drinking, and exercise was adopted from the index currently used in KNHANES. For current smoking, participants who currently smoke with a smoking history of 5 packs (100 cigarettes) were classified as “yes”. For monthly drinking rate, participants who drink at least once a month in the recent 1 year were classified as “yes”. For exercise, participants who did moderate activity (moderate physical effort or breathe somewhat harder than normal) for at least 30 min a day and 5 days during the last 1 week or vigorous activity (high physical effort or breathe much harder than normal) for at least 20 min a day and 3 days during the last 1 week were classified as “yes”. Continuous values were analyzed with Student’s t-test, and categorical values were evaluated with chi-square test for the comparison between sexes. The difference of urine total arsenic and arsenobetaine between gender was analyzed with Mann-Whitney test.

Urine total arsenic, arsenobetaine and total arsenic minus arsenobetaine were classified into four groups according to quartile (overall of 369 participants) to evaluate whether HOMA-β has significant differences among the different groups. The association of urine total arsenic, arsenobetaine, arsenic minus arsenobetaine and fasting glucose, fasting insulin, HOMA-IR and HOMA-β was analyzed with analysis of variance (ANOVA). Bonferroni test was used for post hoc. Because of the significance of sexual discrepancy of general characteristics, every statistical analysis was performed with and without sexual stratification. Then, multivariate analysis was performed for the association between urine arsenobetaine and HOMA-β, which showed a statistically significant association in univariate analysis. HOMA-IR was also analyzed to study pancreatic β-cell function with insulin resistance [15]. In the multivariate analysis, a multiple linear regression method was used, adjusting for factors affecting pancreatic β-cell or diabetes development, such as age [16], smoking [17], drinking [18], body mass index [19], and exercise [20]. Furthermore, blood mercury level, categorized to quartile, was included in the analysis to adjust for effect of seafood intake [21, 22]. Then, analysis was done after stratifying for sex. SPSS 23.00 software (IBM, Chicago, USA) was adopted for statistical analysis.

## Results

### General characteristics of study participants

**Fig. 1:**
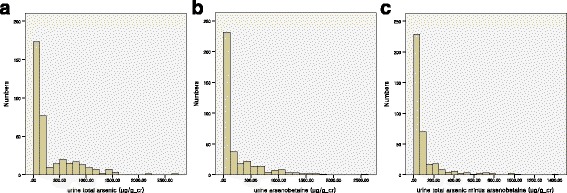
The distribution of (**a**) urine total arsenic, (**b**) arsenobetaine and (**c**) total arsenic minus arsenobetaine among participants

The general characteristics of participants are shown in Table 1. Among a total of 369 participants, 174 were males, and 195 were females. The average age of all participants was 42.31, whereas that of males was 41.54 and that of females was 42.99. The average value of HOMA-β was 122.67 for all participants, 114.92 for men, and 129.59 for women. The median value of urine total arsenic was 136.89 μg/g_Cr for all participants, 143.61 μg/g_Cr for men, and 123.15 μg/g_Cr for women. The median value of urine arsenobetaine was 72.13 μg/g_Cr for all participants, 94.54 μg/g_Cr for men, and 51.55 μg/g_Cr for women, which were statistically significantly different. The median value of urine total arsenic minus arsenobetaine was 43.16 μg/g_Cr for all participants, 46.41 μg/g_Cr for men, and 37.83 for women (Table 2). Six of the participants showed below the detection limit of urine arsenobetaine, two of them were male and four were female. Thirty seven of participants showed negative value on total arsenic minus arsenobetaine. The negative value was included in the group below the 25th percentile, coded to 0.00. The distribution of urine total arsenic, arsenobetaine and total arsenic minus arsenobetaine is shown in Fig. 1.

**Table 1:** General characteristics of the study participants

Variables	Total	Male	Female	*p* value^a^
Number	369	174	195	
Age, years	42.31 ± 15.18^b^	41.54 ± 14.62	42.99 ± 15.67	0.361
Height, cm	164.06 ± 9.31	171.28 ± 6.54	157.62 ± 6.14	<0.001
Weight, kg	62.83 ± 11.69	70.14 ± 10.55	56.3 ± 8.32	<0.001
Waist circumference, cm	79.73 ± 9.64	83.37 ± 9.19	76.48 ± 8.85	<0.001
BMI, kg/cm^2^	23.24 ± 3.22	23.9 ± 3.28	22.66 ± 3.06	<0.001
Fasting plasma glucose, mg/dL	92.35 ± 9.09	94.13 ± 9.51	90.76 ± 8.4	<0.001
Blood insulin, mIU/l	9.28 ± 3.68	9.23 ± 4.07	9.32 ± 3.31	0.828
HOMA-β, %	122.67 ± 57.85	114.92 ± 58.62	129.59 ± 56.41	<0.01
Smoking status^c^				<0.001
Yes	104 (28.2%)	82 (47.1%)	22 (11.3%)	
No	265 (71.8%)	92 (52.9%)	173 (88.7%)	
Alcohol consumption^d^				<0.001
Yes	224 (60.7%)	131 (75.3%)	93 (47.7%)	
No	145 (39.3%)	43 (24.7%)	102 (52.3%)	
Regular excersice^e^				0.065
Yes	106 (28.7%)	58 (33.3%)	48 (24.6%)	
No	263 (71.3%)	116 (66.7%)	147 (75.4%)	
Blood mercury, μg/L	4.461 (2.982, 5.956)^f^	5.465 (3.469, 8.886)	3.880 (2.782, 5.698)	<0.001

^a^ *p* values were from independent t-test and Mann-Whitney test for continous variables and chi-square test for categorical variables

^b^Values are presented as arithmetic mean ± standard deviation

^c^Smoking status was indicated as ‘yes’ for participants who had smoked more than five packs of cigarettes during their life and were currently smoking

^d^Alcohol consumption was indicated as ‘yes’ for participants who consumed at least one glass of alcohol every month over the previous year

^e^Regular exercise was indicated as ‘yes’ when the participant performed mederate or strenuous exercise on a regular basis (for more than 30 min at a time and more than five times per week in the case of moderate exercise; for more than 20 min at a time in the case of strenuous exercise)

^f^Values are presented as median (25th percentile, 75th percentile)

**Table 2:** Urine arsenic and arsenobetaine levels of the study participants

Variables	Total	Male	Female	*p* value^a^
urine total arsenic (μg/g_cr)	136.89 (35.29, 521.39)^b^	143.61 (46.97, 563.24)	123.15 (27.91, 470.27)	0.055
urine arsenobetaine (μg/g_cr)	72.13 (16.64, 325.60)	94.54 (28.46, 369.85)	51.55 (12.40, 232.58)	<0.05
urine total arsenic minus arsenobetaine (μg/g_cr)	43.16 (14.53, 106.36)	46.41 (17.70, 104.80)	37.83 (13.60, 37.83)	0.686

^a^ *p* values are calculated with Mann-Whitney test

^b^Values are presented as median (25th percentile, 75th percentile)

### Differences in fasting insulin and fasting glucose according to urine total arsenic and arsenobetaine

Univariate analysis was performed with ANOVA. Fasting glucose was significantly associated with urine total arsenic level (*p* < 0.05). Post hoc test result showed the difference from the group between the 25–50th percentile and above the 75th percentile. In sexually stratified analysis, no association was found among fasting glucose, fasting insulin and urine total arsenic in both gender (Additional file 1: Table S1).

Fasting glucose was significantly associated with urine arsenobetaine (*p* < 0.001), while fasting insulin was not (*p* = 0.259). The group between the 25–50th percentile showed lowest glucose level. Significant difference was found among the group between the 25–50th percentile, the group between the 50–75th percentile and the group above the 75th percentile. Fasting glucose was also significantly associated with urine arsenobetaine in female (*p* < 0.05). The group between the 25–50th percentile was significantly different from the group above the 75th percentile (Table 3). No association was shown between fasting glucose, insulin and urine total arsenic minus arsenobetaine (Additional file 2: Table S2).

**Table 3:** Univariate analysis of the relationship between fasting glucose, insulin and urine arsenobetaine

Urine arsenobetaine (μg/g_cr)	Total						Male					Female					
	Number	Fasting glucose (mg/dL)	*p* value^a^	Post Hoc^b^	Insulin (mIU/L)	*p* value	Number	Fasting glucose (mg/dL)	*p* value	Insulin (mIU/L)	*p* value	Number	Fasting glucose (mg/dL)	*p* value	Post hoc	Insulin (mIU/L)	*p* value
1st quartile (Undetectable-16.36)	92	91.38 ± 8.56^c^	<0.001	1.000	9.90 ± 4.04	0.259	34	92.54 ± 8.65	0.175	10.88 ± 5.20	0.055	58	90.67 ± 8.50	<0.05	0.702	9.30 ± 3.04	0.947
2nd quartile (16.91–70.70)	92	89.84 ± 8.22		Reference	9.15 ± 3.53		47	92.56 ± 9.93		8.59 ± 3.29		45	88.24 ± 6.61		Reference	9.47 ± 3.66	
3rd quartile (71.57–321.71)	92	93.39 ± 8.44		<0.05	8.85 ± 3.46		45	94.03 ± 8.84		8.73 ± 3.60		47	92.29 ± 7.73		1.00	9.04 ± 3.26	
4th quartile (324.19–2620.78)	93	94.75 ± 10.31		<0.001	9.21 ± 3.64		48	96.55 ± 10.39		9.08 ± 3.99		45	92.91 ± 10.02		<0.05	9.34 ± 3.29	

^a^ *p* values are calculated with ANOVA

^b^Post hoc by Bonferroni test

^c^Values are presented as arithmetic mean ± standard deviation

### The relationship among urine total arsenic, urine arsenobetaine, and HOMA index

Univariate analysis was performed with ANOVA, using the percentile-based categorized value of urine total arsenic and urine arsenobetaine as independent variables and HOMA-IR, HOMA-β as a dependent variable. HOMA-IR (*p* = 0.184) and HOMA-β (*p* = 0.476) were not significantly associated with total urine arsenic (Additional file 3: Table S3). HOMA-β showed significant differences according to urine arsenobetaine level (*p* < 0.01). Post hoc showed the significant difference between the group below the 25th percentile, between the 50–75th percentile and above the 75th percentile. HOMA-IR did not showed significant difference (*p* = 0.41). In sexually stratified analysis, only HOMA-β was significantly different based on urine arsenobetaine only in males (*p* < 0.05), while HOMA-IR did not (Table 3). Post hoc test was done for HOMA-β and arsenobetaine. The lowest quartile group shows significantly higher HOMA-β than the third and fourth quartile group (Table 4). No significant association was shown between HOMA-β, HOMA-IR and urine total arsenic minus arsenobetaine (Additional file 4: Table S4).

**Table 4:** Univariate analysis of the relationship between HOMA index and urine arsenobetaine

Urine arsenobetaine (μg/g_cr)	Total						Male						Female				
	Number	HOMA-β (%)	*p* value^a^	Post hoc^b^	HOMA-IR	*p* value	Number	HOMA-β (%)	*p* value	Post hoc	HOMA-IR	*p* value	Number	HOMA-β (%)	*p* value	HOMA-IR	*p* value
1st quartile (Undetectable-16.36)	92	135.97 ± 68.01^c^	<0.01	Reference	2.24 ± 1.00	0.41	35	143.74 ± 79.33	<0.01	Reference	2.50 ± 1.27	0.146	57	131.2 ± 60.29	0.064	2.09 ± 0.76	0.966
2nd quartile (16.91–70.70)	92	132.12 ± 60.6		1.000	2.04 ± 0.90		34	112.46 ± 49.85		0.146	1.99 ± 0.89		58	143.64 ± 63.7		2.08 ± 0.91	
3rd quartile (71.57–321.71)	92	110.76 ± 48.41		<0.05	2.05 ± 0.97		58	109.19 ± 53.81		<0.05	2.04 ± 0.90		34	113.97 ± 38.01		2.08 ± 0.87	
4th quartile (324.19–2620.78)	93	111.76 ± 48.34		<0.05	2.18 ± 0.94		47	102.29 ± 45.38		<0.01	2.16 ± 1.04		46	121.44 ± 49.84		2.15 ± 0.85	

^a^ *p* values are calculated with ANOVA

^b^Post hoc by Bonferroni test

^c^Values are presented as arithmetic mean ± standard deviation

In multivariate analysis of urine arsenobetaine and HOMA-β, the value of HOMA-β in the group below the 25th percentile of urine arsenobetaine was significantly higher than that in the group between the 50–75th percentile. Among male participants, the value of HOMA-β in the group below the 25th percentile of urine arsenobetaine was significantly higher than that in the group between the 25–50th percentile and 50–75th percentile whereas HOMA-IR was not significant difference; however, there was no significant difference of HOMA-β and HOMA-IR among the groups for female participants (Table 5).

**Table 5:** Multiple linear regression analysis of the relationship between HOMA-β, HOMA-IR and urine arsenobetaine

Variables	Total				Male				Female			
	HOMA-β (%)		HOMA-IR		HOMA-β (%)		HOMA-IR		HOMA-β (%)		HOMA-IR	
	Coefficient (95% C.I)	*p* value	Coefficient (95% C.I)	*p* value	Coefficient (95% C.I)	*p* value	Coefficient (95% C.I)	*p* value	Coefficient (95% C.I)	*p*-value	Coefficient (95% C.I)	*p* value
Urine arsenobetaine (μg/g_cr)												
1st quartile (Undetectable-16.36)	Reference		Reference		Reference		Reference		Reference		Reference	
2nd quartile (16.91–70.70)	−9.613 (−24.490, 5.264)	0.205	−0.208(−0.449, 0.033)	0.091	−30.043(−53.954, −6.133)	<0.05	−0.405(−0.835, −0.025)	0.065	5.394(−13.276, 24.064)	0.571	−0.059(−0.329, 0.211)	0.668
3rd quartile (71.57–321.71)	−17.796(−33.257, −2.334)	<0.05	−0.219(−0.469, 0.031)	0.086	−22.382(−44.023, −0.742)	<0.05	−0.343(−0.732, 0.046)	0.084	−12.575(−35.104, 9.954)	0.274	−0.071(−0.397, 0.254)	0.667
4th quartile (324.19–2620.78)	−8.368(−24.891, 8.056)	0.318	−0.038(−0.304,0.228)	0.779	−14.950(−39.251, 9.351)	0.228	−0.027(−0.464, 0.410)	0.902	−3.857(−25.647, 17.932)	0.729	−0.055(−0.370, 0.259)	0.731
Age (years)	01.117(−1.486,0.748)	<0.001	−0.004(−0.010, 0.002)	0.172	−1.211(−1.752, −0.671)	<0.001	−0.006(−0.015, 0.004)	0.258	−1.052(−1.558, −0.546)	<0.001	−0.003(−0.011, 0.004)	0.376
BMI (kg/cm^2^)	5.188 (3.523, 6.853)	<0.001	0.136 (0.109, 0.163)	<0.001	5.465 (3.075,7.855)	<0.001	0.149 (0.106, 0.192)	<0.001	5.565 (3.184, 7.946)	<0.001	0.132 (0.097, 0.166)	<0.001
Smoking^a^	−10.452(−22.42, 1.515)	0.087	−0.114(−0.308, 0.080)	0.248	−11.827(−27.403,3.750)	0.137	−0.251(−0.531, 0.029)	0.079	8.524 (−14.362, 31.410)	0.465	0.242(−0.088, 0.572)	0.151
Alcohol consumption^b^	−14.864(−26.291, −3.436)	<0.05	0.065(−0.120, 0.250)	0.492	−18.557(−37.121, 0.006)	0.050	0.077(−0.256, 0.411)	0.650	−9.699(−24.618, 5.219)	0.203	0.045(−0.170, 0.261)	0.680
Regular exercise^c^	−4.067(−15.791, 7.657)	0.497	−0.137(−0.327, 0.053)	0.157	−2.490(−18.472, 13.492)	0.760	−0.031(−0.318, 0.256)	0.833	−4.731(−21.725, 12.264)	0.585	−0.260(−0.505, −0.015)	<0.05
Blood mercury (μg/L)												
1st quartile (1.188–2.977)	Reference		Reference		Reference		Reference		Reference		Reference	
2nd quartile (2.987–4.598)	15.686 (1.036,30.337)	<0.05	−0.046(−0.283, 0.191)	0.705	−0.415(−25.210, 24.381)	0.974	−0.264(−0.710, 0.182)	0.246	27.067 (8.938, 45.197)	<0.01	0.108(−0.153, 0.370)	0.417
3rd quartile (4.625–6.969)	5.642(−10.252, 21.536)	0.487	0.023(−0.235, 0.280)	0.862	−2.752(−27.702, 22.197)	0.829	−0.388(−0.836, 0.061)	0.090	11.404(−9.568, 32.377)	0.287	0.304 (0.001, 0.607)	<0.05
4th quartile (6.997–37.654)	−15.172(−32.179, 1.835)	0.08	−0.165(−0.440, 0.110)	0.240	−27.387(−52.474, −2.300)	<0.05	−0.524(−0.975, −0.073)	<0.05	3.667(−21.177, 28.512)	0.772	0.178(−0.181, 0.536)	0.332

*Abbreviations*: *BMI* body mass index, *C.I* confidence interval

^a^Smoking status was indicated as ‘yes’ for participants who had smoked more than five packs of cigarettes during their life and were currently smoking

^b^Alcohol consumption was indicated as ‘yes’ for participants who consumed at least one glass of alcohol every month over the previous year

^c^Regular exercise was indicated as ‘yes’ when the participant performed mederate or strenuous exercise on a regular basis (for more than 30 min at a time and more than five times per week in the case of moderate exercise; for more than 20 min at a time in the case of strenuous exercise)

## Discussion

This epidemiological study investigated the association between β-cell function (a predictive factor of diabetes development) and the organic arsenic arsenobetaine (an agent less toxic to humans). Urine arsenobetaine was revealed to be associated with HOMA-β in the normal population (without diabetes), especially in males, even after adjusting for factors affecting diabetes and β-cell function, while no association was shown with HOMA-IR which is associated with insulin resistance.

Despite several conflicting results, arsenic has been reported to be correlated with diabetes in many epidemiological studies [6, 9, 23–31], and arsenic can affect insulin resistance and β-cell function [7]. In some experimental studies, arsenite was found to induce oxidative stress in pancreatic β-cells and reduce insulin transcription and secretion [32, 33], caused by the altered expression of essential genes such as the Pdx1 gene [34, 35] and insulin gene [36] for pancreas development, insulin production, and glucose homeostasis maintenance. Moreover, a study reported that arsenite can affect reactive oxygen species (ROS) production to intervene glucose uptake [37]. Arsenate also reported to affect on glucose homeostasis and ATP-dependent insulin secretion, by forming ADP-arsenite and glucose-6-arsenate [38]. However, DMA or MMA do not perturb phosphate metabolism [38].

This study demonstrated the association among β-cell function and arsenobetaine, one of the organic species of arsenic, by revealing the correlation between urine arsenobetaine and HOMA-β in the normal population (without diabetes). HOMA-β was used in this study as an index reflecting the function of pancreatic β-cells [4]. Also, previous antegrade studies revealed that HOMA-β reduction is an important factor for predicting diabetes development [39–41].

Previous studies showed that arsenobetaine is hardly metabolized in the body and rapidly excreted [42], and the lethal dose (LD) 50 of arsenobetaine is over 10,000 mg/kg [43]. Various studies in animals reported that arsenobetaine is not mutagenic, not cytotoxic, and does not have transforming activity [44]. Following those results, arsenobetaine has been categorized as non-toxic. Therefore, typical studies related to the toxic effect of arsenic have focused on inorganic arsenic, and the sum of inorganic-related species (arsenate, arsenite, dimethylarsinic acid (DMA), and monomethylarsonic acid (MMA)) has been adopted as a biological index of arsenic exposure [45]. Moreover, previous epidemiological studies excluded the indigestion of marine products, which is a main source of arsenobetaine exposure, and investigated the association between diabetes and urine total arsenic after subtracting the value of urine arsenobetaine; as a result, conflicting results were obtained regarding arsenobetaine in a statistical model [5, 9].

However, one study showed the continuous urine excretion of 0.2 ~ 12.2 μg/L of arsenobetaine in several participants 12 days after complete restriction of arsenobetaine indigestion, which suggested that arsenobetaine may be a metabolite of other arsenic species such as inorganic arsenic or DMA or accumulated arsenobetaine is released slowly [46]. Moreover, earlier studies did not confirm whether arsenobetaine can be metabolized in the actual digestive process because most of those studies used only drinking solution [47, 48]. Arsenobetaine can be transformed into toxic forms, such as DMA, by intestinal microorganisms in the actual digestive process when it is absorbed through food. An in vivo experiment demonstrated that arsenobetaine can be degraded into DMA, dimethylarsinoylacetic acid (DMAA), and trimethylarsine oxide (TMAO) in microcosm inoculated with human fecal matter, suggesting that the biocatalytic capability for arsenobetaine exists in the human gastrointestinal tract [12]. A study on humans showed that after ingestion of prawns containing 98.8% arsenobetaine, 0.14% DMA, and 0.96% inorganic acid, 3–5% arsenobetaine was excreted as inorganic arsenic, MA, and DMA [49]. Another study suggests that methylarsonic acid (MA) and DMA can be demethylated in the intestine, thus producing arsenate [50]. Taken together, it appears that arsenobetaine may accumulate in the body and become toxic or may metabolize into other toxic substances, such as arsenate, leading to diabetogenic toxicity; however, the mechanisms involved remain unclear. Further research should be conducted to elucidate the mechanism of arsenobetaine metabolism, especially in the human digestive system. Our result showed that arsenobetaine may have a diabetogenic effect on humans. Further studies are needed to determine whether arsenobetaine is a biomarker with chronic toxicity through metabolism or produced from other toxic arsenic species and to investigate the association among β-cell function, arsenobetaine, and the development of diabetes.

Since general characteristics exhibited sexual discrepancy, sexually stratified analysis was performed. The results of this study were significant only for male participants. The female gender is known to be a protective factor against diabetes. Epidemiologic studies on humans have revealed that females have a lower prevalence of diabetes until the time of menopause [51] despite having less skeletal muscle mass and more adipose mass [52]. Estradiol has been suggested as the protective factor against diabetes in animals and humans [52]. Pancreatic β-cells have ERα receptors, which are associated with glucose metabolism, insulin secretion, and β-cell survival [53]. Various animal studies have been performed to evaluate the effect of estrogen on pancreatic β-cell physiology. One study showed that the upregulation of pancreatic β-cell insulin can be initiated with ERα activation by the endocrine disruptor bisphenol-A as well as ERα and ERβ agonists in mice [54]. Le May et al. [55] suggested that ERα plays a key role in pancreatic β-cell survival from oxidative stress. The metabolic pathway of arsenic is also known to be affected by sex, as indicated in several studies [56–58]. Previous epidemiological studies showed that sex can affect the correlation between diabetes development and arsenic; in the group exposed to inorganic arsenic, men who had a higher incidence of diabetes were below 40, whereas women who had a higher incidence were above 40, especially after menopause [27, 59], which suggest that estrogen status can affect the mechanism of arsenic on developing diabetes. Other studies reported that the pathophysiology of developing diabetes caused by arsenic is affected by estrogen and estrogen receptor, and an experiment on mice showed that inorganic arsenic can influence glucose regulation in the estrogen-deficient state [60, 61]. These findings suggest that the female gender may contribute to the protective effect against arsenic in β-cell function, which can be the cause of sexual discrepancy in our results. Consistently, our study found that the male population appears to be a more sensitive group to arsenobetaine exposure. However, there have been no studies regarding the effect of sex and sexual hormones on the metabolism and toxic effect of arsenobetaine. Further studies are needed to determine whether sex affects the metabolic pathway of arsenobetaine and diabetes development.

In our study, the effect of arsenobetaine on pancreatic β-cell function appears to be non-linear. Rather, arsenobetaine appears to have a hormesis-like effect. In multivariate analysis, the HOMA- β value of the group over the 75th percentile group was not significantly different from that of the 0–25th group, while the value of the 25–50th percentile group and the 50–75th group was significantly lower than that of the 0–25th percentile group. Arsenobetaine may have both negative and positive effects on pancreatic β-cell function through various pathways. Arsenobetaine is an arsenic analog of trimethylglycine, known as betaine. Arsenobetaine is also an osmolyte analog that is accumulated in tissues by the transport system [62]. Organic osmolyte functions to protect cells from stress [63] via various pathways associated with regulation of cellular hydration state [64]. Pancreatic β-cells are modulated by intracellular ATP concentration and volume-sensitive anion-selective channels. Accumulation of intracellular osmolyte is responsible for pancreatic β-cell swelling, associated with anion channel activation and anion efflux [65, 66]. However, although theoretically possible, it has not been proven. Further research should be conducted to elucidate the mechanism of arsenobetaine exposure in human pancreatic β-cells.

There are several limitations in this study. First, this cross-sectional study was unable to identify a causal relationship of arsenobetaine with pancreatic β-cell function. Second, urine arsenobetaine is easily affected by indigestion of marine products and seaweed, though we were unable to directly analyze the recent intake amount of marine products. Seafood intake is also known to be associated with diabetes [67, 68]. However, we included blood mercury level as an index of seafood intake [21, 22] to adjust for the effect of seafood intake and minimize the effect of seafood intake. Third, we hypothesized that arsenobetaine has diabetogenic toxicity from being metabolized to the inorganic arsenic, which is known to be toxic and diabetogenic. We calculated the ‘total arsenic minus arsenobetaine’ to evaluate the non-arsenobetaine portion of arsenic and glucose metabolism. Although total arsenic minus arsenobetaine includes arsenocholine, arsenosugar, and arsenolipid metabolism and is not an ideal marker of inorganic arsenic [5, 69], it has been used as a marker of inorganic acid in several studies [9, 70, 71]. Total arsenic minus arsenobetaine did not demonstrate a significant association with HOMA index. Arsenate and arsenite, which are known to be directly associated with pancreatic β-cell toxicity and glucose metabolism, could not be appropriately evaluated because 296 (80.2%) of arsenate, 229 (62.1%) of arsenite were below the detection limit value. Methylated arsenic also could not be evaluated sufficiently; 237 (64.2%) of MMA, 37 (10.0%) of DMA were below the limit value. Despite these limitations, this study demonstrated that HOMA-β value, which indicates pancreatic β-cell function, is associated with urine arsenobetaine, especially in males. We suggest that further studies on arsenic and diabetes development should treat urine arsenobetaine and indigestion of marine products as independent variables that can affect the pancreatic β-cell function and developmental process of diabetes instead of excluding or adjusting for them. Moreover, further investigation is needed to assess the metabolism and chronic toxicity of arsenobetaine.

## Conclusion

This study revealed the association between urine arsenobetaine and pancreatic β-cell function assessed by HOMA-β in the normal population (without diabetes), especially in males, despite adjusting for factors affecting pancreatic β-cell function and diabetes.

## Additional files

Additional file 1: Table S1. Univariate analysis of the relationship between fasting glucose, insulin and urine total arsenic. (DOCX 20 kb)

Additional file 2: Table S2. Univariate analysis of the relationship between fasting glucose, insulin and urine total arsenic minus arsenobetaine. (DOCX 18 kb)

Additional file 3: Table S3. Relationship between HOMA index and urine total arsenic. (DOCX 17 kb)

Additional file 4: Table S4. Univariate analysis of the relationship between HOMA index and urine total arsenic minus arsenobetaine. (DOCX 17 kb)

## Abbreviations

ANOVA: Analysis of variance
C.I: Confidence interval
DMA: Dimethyl arsenic acid
DMAA: Dimethylarsonylacetic acid
HOMA: Homeostasis model assessment
ICP-MS: Inductively coupled plasma-mass spectrometry
KNHANES: Korea National Health and Nutrition Examination Survey
MA: Methylarsonic acid
MMA: Monomethylarsonic acid
ROS: Reactive oxygen species
TMAO: Trimethylarsine oxide
