# Supplementary material for: Association of arsenobetaine with beta-cell function assessed by homeostasis model assessment (HOMA) in nondiabetic Koreans: data from the fourth Korea National Health and Nutrition Examination Survey (KNHANES) 2008-2009
Source: Ann Occup Environ Med. 2017 Jul 10;29:31. doi: 10.1186/s40557-017-0181-0 (PMC5504790; doi:10.1186/s40557-017-0181-0)
Supplement: Supplementary file 1 [file 40557_2017_181_MOESM1_ESM.docx]

Supplementary Material, Table 1.

Univariate analysis of the relationship between fasting glucose, insulin and urine total arsenic

	Total						Male					Female				
Urine total arsenic (μg/g_cr)	Number	Fasting glucose (mg/dL)	P value[a]	Post Hoc[b]	Insulin (mIU/L)	P value	Number	Fasting glucose (mg/dL)	P value	Insulin (mIU/L)	P value	Number	Fasting glucose (mg/dL)	P value	Insulin (mIU/L)	P value
1st quartile (2.51-35.24)	92	91.84±8.22[c]	<0.05	1.000	9.69±4.04	0.502	34	94.06±9.01	0.276	10.58±5.29	0.195	58	90.53±7.51	0.121	9.17±3.03	0.68
2nd quartile (35.34-134.88)	92	91.10±8.49		reference	9.29±4.03		47	92.57±8.56		9.03±4.13		45	89.56±8.23		9.55±3.95	
3rd quartile (136.89-511.27)	92	91.59±8.73		1.000	8.86±3.10		45	93.51±9.81		8.79±3.18		47	89.74±7.20		8.92±3.01	
4th quartile (517.58-2731.29)	93	94.84±10.39		<0.05	9.26±3.68		48	96.27±10.35		8.88±3.69		45	93.31±10.34		9.67±3.30	

[a]P values are calculated with ANOVA

[b]Post hoc by Bonferroni test

[c]Values are presented as arithmetic mean ± standard deviation
