# Supplementary material for: Association of arsenobetaine with beta-cell function assessed by homeostasis model assessment (HOMA) in nondiabetic Koreans: data from the fourth Korea National Health and Nutrition Examination Survey (KNHANES) 2008-2009
Source: Ann Occup Environ Med. 2017 Jul 10;29:31. doi: 10.1186/s40557-017-0181-0 (PMC5504790; doi:10.1186/s40557-017-0181-0)
Supplement: Supplementary file 2 [file 40557_2017_181_MOESM2_ESM.docx]

Supplementary Material, Table 2.

Univariate analysis of the relationship between fasting glucose, insulin and urine total arsenic minus arsenobetaine

	Total					Male					Female				
Urine total arsenic minus arsenobetaine (μg/g_cr)	Number	Fasting glucose (mg/dL)	P value[a]	Insulin (mIU/L)	P value	Number	Fasting glucose (mg/dL)	P value	Insulin (mIU/L)	P value	Number	Fasting glucose (mg/dL)	P value	Insulin (mIU/L)	P value
1st quartile (0.00[c]-10.13)	92	92.33±8.52[b]	0.508	9.40±3.45	0.88	39	94.77±8.15	0.413	9.81±4.22	0.208	53	90.53±8.05	0.715	9.10±2.83	0.736
2nd quartile (10.26-33.64)	93	92.87±8.83		9.42±4.17		48	95.25±9.14		9.65±4.74		45	90.33±7.81		9.17±3.51	
3rd quartile (34.48-98.98)	92	91.17±8.66		9.26±3.17		47	92.17±9.66		9.31±3.78		45	90.13±7.44		9.21±3.70	
4th quartile (99.60-1428.77)	92	92.35±9.09		9.02±3.35		40	94.45±10.94		8.07±3.23		52	91.90±9.71		9.76±3.28	

[a]P values are calculated with ANOVA

[b]Values are presented as arithmetic mean ± standard deviation

[c]Negative values were coded to 0.00
