# Supplementary material for: Association of arsenobetaine with beta-cell function assessed by homeostasis model assessment (HOMA) in nondiabetic Koreans: data from the fourth Korea National Health and Nutrition Examination Survey (KNHANES) 2008-2009
Source: Ann Occup Environ Med. 2017 Jul 10;29:31. doi: 10.1186/s40557-017-0181-0 (PMC5504790; doi:10.1186/s40557-017-0181-0)
Supplement: Supplementary file 3 [file 40557_2017_181_MOESM3_ESM.docx]

lementary Material, Table 3.

ionship between HOMA index and urine total arsenic

Urine total arsenic (μg/g_cr)	Total					Male					Female				
	Number	HOMA-β (%)	P value[a]	HOMA-IR	P value	Number	HOMA-β (%)	P value	HOMA-IR	P value	Number	HOMA-β (%)	P value	HOMA-IR	P value
1st quartile (2.51-35.24)	92	128.69±61.09[b]	0.184	2.21±1.00	0.476	34	131.73±76.55	0.164	2.47±1.31	0.271	58	126.91±50.57	0.53	2.05±0.78	0.474
2nd quartile (35.34-134.88)	92	128.38±63.64		2.11±1.03		47	117.32±59.13		2.08±1.03		45	139.93±66.73		2.14±1.05	
3rd quartile (136.89-511.27)	92	121.25±56.27		2.01±0.77		45	113.01±51.92		2.04±0.85		47	129.13±59.63		1.98±0.71	
4th quartile (517.58-2731.29)	93	112.48±48.79		2.19±0.92		48	102.43±47.07		2.13±0.98		45	123.2±48.82		2.24±0.86	

es are calculated with ANOVA

ed as arithmetic mean ± standard deviation
