# Supplementary material for: Association of arsenobetaine with beta-cell function assessed by homeostasis model assessment (HOMA) in nondiabetic Koreans: data from the fourth Korea National Health and Nutrition Examination Survey (KNHANES) 2008-2009
Source: Ann Occup Environ Med. 2017 Jul 10;29:31. doi: 10.1186/s40557-017-0181-0 (PMC5504790; doi:10.1186/s40557-017-0181-0)
Supplement: Supplementary file 4 [file 40557_2017_181_MOESM4_ESM.docx]

Supplementary Material, Table 4.

Univariate analysis of the relationship between HOMA index and urine total arsenic minus arsenobetaine

Urine total arsenic minus arsenobetaine (μg/g_cr)	Total					Male					Female				
	Number	HOMA-β (%)	P value[a]	HOMA-IR	P value	Number	HOMA-β (%)	P value	HOMA-IR	P value	Number	HOMA-β (%)	P value	HOMA-IR	P value
1st quartile (0.00-10.13)	92	123.45±55.05[b]	0.834	2.15±0.87	0.939	39	117.13±61.55	0.281	2.32±1.08	0.285	53	128.11±49.82	0.963	2.03±0.66	0.609
2nd quartile (10.26-33.64)	93	123.10±66.21		2.17±1.04		48	114.94±63.07		2.28±1.18		45	131.81±69.03		2.05±0.87	
3rd quartile (34.48-98.98)	92	125.94±54.21		2.11±0.96		47	125.15±61.35		2.13±0.97		45	126.77±46.29		2.08±0.97	
4th quartile (99.60-1428.77)	92	118.19±55.68		2.09±0.88		40	100.71±44.51		1.91±0.87		52	131.63±59.95		2.23±0.86	

[a]P values are calculated with ANOVA

[b]Values are presented as arithmetic mean ± standard deviation
